# Combined Use of High-Sensitive Cardiac Troponin, Copeptin, and the Modified HEART Score for Rapid Evaluation of Chest Pain Patients

**DOI:** 10.1155/2018/9136971

**Published:** 2018-11-12

**Authors:** Beata Morawiec, Brygida Przywara-Chowaniec, Piotr Muzyk, Mariusz Opara, Lam Ho, Lui Chun Tat, Olivier Muller, Ewa Nowalany-Kozielska, Damian Kawecki

**Affiliations:** ^1^2nd Department of Cardiology, School of Medicine with the Division of Dentistry in Zabrze, Medical University of Silesia, Katowice, Poland; ^2^Department of Internal Medicine with Cardiology, Tuen Mun Hospital, Hong Kong; ^3^Department of Cardiology, University Hospital (CHUV), Lausanne, Switzerland

## Abstract

**Background:**

Clinical short-term risk stratification is a recommended approach in patients with chest pain and possible acute myocardial infarction (AMI) to further improve high safety of biomarker-based rule-out algorithms. The study aim was to assess clinical performance of baseline concentrations of high-sensitivity cardiac troponin T (hs-TnT) and copeptin and the modified HEART score (mHS) in early presenters to the emergency department with chest pain.

**Methods:**

This cohort study included patients with chest pain with onset maximum of 6 h before admission and no persistent ST-segment elevation on electrocardiogram. hs-TnT, copeptin, and the mHS were assessed from admission data. The diagnostic and prognostic value for three baseline rule-out algorithms: (1) single hs-TnT < 14 ng/l, (2) hs-TnT < 14 ng/l/mHS ≤ 3, and (3) hs-TnT < 14 ng/l/mHS ≤ 3/copeptin < 17.4 pmol/l, was assessed with sensitivity and negative predictive value. Primary diagnostic endpoint was the diagnosis of AMI. Prognostic endpoint was death and/or AMI within 30 days.

**Results:**

Among 154 enrolled patients, 44 (29%) were classified as low-risk according to the mHS; AMI was diagnosed in 105 patients (68%). For ruling out AMI, the highest sensitivity and NPV from all studied algorithms were observed for hs-TnT/mHS/copeptin (100%, 95% CI 96.6–100, and 100%, 95% CI 75.3–100). At 30 days, the highest event-free survival was achieved in patients stratified with hs-TnT/mHS/copeptin algorithm (100%) with 100% (95% CI 75.3–100) NPV and 100% (95% CI 96.6–100) sensitivity.

**Conclusions:**

The combination of baseline hs-TnT, copeptin, and the mHS has an excellent sensitivity and NPV for short-term risk stratification. Such approach might improve the triage system in emergency departments and be a bridge for inclusion to serial blood sampling algorithms.

## 1. Introduction

The quality of the management of patients with acute chest pain in the emergency department is constantly improving; however, high safety is achieved at the expense of efficacy. High-sensitivity cardiac troponin (hs-Tn) has been shown to carry high diagnostic value in this setting [1–6]. Serial sampling in short time intervals has been studied and validated to increase the safety and efficacy of early discharge without the need for further testing [1, 7–10]. Copeptin and clinical scores were tested and are recommended to further increase high sensitivity of hs-Tn regarding diagnostic and prognostic evaluation [11–16]. From the variety of risk scores, the HEART score combines the most common characteristics used for routine clinical evaluation and assessment of the probability of cardiac origin of chest pain and acute coronary syndrome. Easy to assess at bedside in baseline examination, the HEART score improved the performance of standard cardiac troponin; however, high safety achieved with such combination was balanced by time delay to serial measurements of biomarkers [11]. Originally composed of history, electrocardiographic findings, age, risk factors, and standard markers of cardiac injury, the HEART score was modified by using high-sensitive troponin assays. Recent studies controvert the value from assessment of the modified HEART score (mHS) in the era of hs-Tn [17]. To date, there is no data reporting on safety of a multimarker strategy and the HEART score at baseline and after introduction of high-sensitivity assays. We therefore aimed to assess the clinical performance of the mHS with baseline concentrations of hs-Tn and copeptin in early presenters to the emergency department with chest pain and suspected acute coronary syndrome.

## 2. Materials and Methods

### 2.1. Study Design

This is a cohort, cross-sectional study, primary designed to evaluate the role of copeptin in rapid evaluation of patients with acute chest pain in the emergency department (COPeptin for Acute Coronary Syndrome/COPACS/study).

Details on the design and the chart of the study were widely described previously [9]. In brief, consecutive patients presenting to the emergency department of the 2nd Department of Cardiology, Zabrze, Medical University of Silesia, Katowice, Poland, were enrolled. Inclusion criteria were chest pain of a minimum of 5-minute duration with beginning during the last 6 hours. Patients were excluded in the presence of persistent ST-segment elevation in an electrocardiogram (ECG) at admission or major conditions with proved influence on copeptin elevation (e.g., end-stage renal disease, sepsis, anaemia, and hyponatremia). The study protocol conforms to the ethical guidelines of the Declaration of Helsinki and was approved by the Ethical Committee of the Medical University of Silesia (decision no. KNW/0022/KB1/187/11). All patients gave their written informed consent before inclusion to the study. The study registration number is ISRCTN14112941 (http://www.isrctn.com). The design of the study, data gathering, and analysis were conducted according to the STARD guidelines for studies of diagnostic/prognostic accuracy.

### 2.2. Clinical Assessment and Diagnosis

After inclusion, each patient underwent initial clinical examination which included physical examination, 12-lead ECG, echocardiographic examination, and standard laboratory tests. hs-TnT and copeptin were measured at admission. hs-TnT was afterwards measured at six hours and repeated according to clinical indications. As per study design, copeptin was double-blinded until final adjudication of the diagnosis. hs-TnT was measured routinely in clinical care according to current ESC guidelines for management of patients with ACS and was recorded as such for analysis.

Initial diagnosis was set by the emergency physician and was verified by a supervisor cardiologist based on available data and the ESC guidelines [14]. Further, all included patients underwent routine diagnostic and therapeutic procedures as indicated in the ESC guidelines for non-ST-segment elevation ACS [14] and according to the study design [9].

Final diagnosis of non-ST-elevation myocardial infarction (NSTEMI), unstable angina (UA), or other causes of chest pain was based on independent opinions of two cardiologists, after analysis of all available data and tests gathered during the hospital stay. In case of incoherence of their diagnosis, the opinion of a third cardiologist was conclusive.

### 2.3. Investigational Laboratory Measurements

Copeptin was measured once, at admission, in plasma from the blood sample collected to tubes containing potassium ethylenediaminetetraacetic acid (EDTA) managed according to the instructions of the manufacturer of the test using the BRAHMS Copeptin KRYPTOR kit on BRAHMS KRYPTOR compact plus analyser (BRAHMS GmbH, Hennigsdorf, Germany)—detection limit 4.8–500 pmol/l, 20% coefficient of variation (CV) at 12 pmol/l, and the 97.5th percentile for healthy population 17.4 pmol/l. According to the general rule for the optimal cutoff for a marker at the 99th percentile of healthy population, copeptin was regarded as positive when ≥17.4 pmol/l, following available information provided by the manufacturer on the most compliant value (97.5th percentile) to that recommended in the guidelines [14, 18]. Rule-out zone for copeptin was defined accordingly at less than 17.4 pmol/l.

Cardiac troponin T was measured at admission, after six hours, and at further time points according to discretion of the treating physician, in plasma from a blood sample collected to tubes containing EDTA, with a high-sensitive assay (Elecsys Troponin T hs STAT kit on cobas e 411 analyser, Roche Diagnostics GmbH, Mannheim, Germany) with high-sensitive electrochemiluminescence method (limit of detection 3–10000 ng/l, 99th percentile for healthy population 14 ng/l (95% CI 12.7–24.9 ng/l), and 10% CV of 13 ng/l). hs-TnT rule-out zone was defined as less than 14 ng/l, according to the manufacturer indications and current guidelines [14].

### 2.4. The Modified HEART Score

The mHS was assessed in all patients based on data collected at admission. As previously described [19, 20], the score included the following variables: age, ECG pattern, risk factors, and clinical presentation. The HEART score originally included multiplication of upper limit of standard cardiac troponin as one of the variables, whereas the idea of the mHS is to combine clinical characteristics used previously with current biomarker algorithms and high-sensitive assays.

Each element of the mHS was assigned 0–2 points according to the type or severity of corresponding risk (Table 1). Patients were considered low-risk for adverse events if the mHS was less than or equal to 3, as previously recommended [12, 13, 21].

### 2.5. Endpoints

Per study design, follow-up was carried on at 30 days and one year in a phone call with the patient, relatives, or primary care physician. At one year, during a visit in the outpatient unit, the following data were gathered: CCS and NYHA class, the occurrence of endpoints (major adverse cardiac events/MACE/composed of death, nonfatal acute myocardial infarction/AMI/UA, repeated revascularization, and stroke), echocardiogram with the assessment of left ventricular ejection fraction, and blood draw for N-terminal pro-B-type natriuretic peptide (NT-proBNP).

For current analysis, primary diagnostic endpoint was the diagnosis of AMI. Primary prognostic endpoint was defined as death of cardiovascular origin and/or AMI within 30 days from admission. Secondary prognostic analysis was performed at one year.

### 2.6. Statistical Analysis

Data were checked for normality of distribution with Shapiro-Wilk test. Continuous variables are presented as mean (standard deviation (SD)) or median (interquartile range (IQR)) and were compared with Student *t*-test or Mann-Whitney test, depending on the distribution. Categorical variables are presented as *n*, %, and were compared with chi-square test. Diagnostic and prognostic value was assessed for three rule-out algorithms: (1) single baseline hs-TnT concentration < 14 ng/l, (2) baseline hs-TnT concentration < 14 ng/l and the mHS ≤ 3, and (3) baseline hs-TnT concentration < 14 ng/l, the mHS ≤ 3, and baseline copeptin concentration < 17.4 pmol/l. Safety of the algorithms was assessed with sensitivity and negative predictive value (NPV); clinical accuracy of the algorithms was defined as the proportion of true rule-out and rule-in rates. Kaplan-Meier curves for all three algorithms were used to depict event-free survival at 30 days. The influence of biomarkers and the mHS on the occurrence of endpoints was calculated in Cox proportional hazard regression model. Statistics were performed with IBM SPSS Statistics version 22.0 (SPSS Inc., Chicago, IL) and GraphPad Prism, version 6.00 (GraphPad, La Jolla, California, USA).

## 3. Results

### 3.1. General Characteristics of Patients

**Figure 1:**
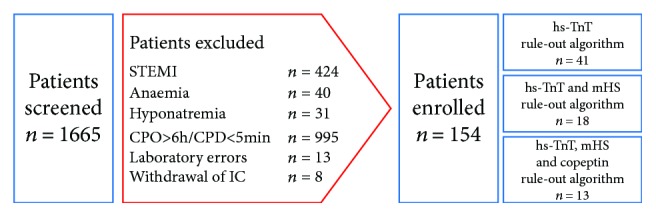
Study chart. STEMI: ST-segment elevation myocardial infarction; CPO: chest pain onset; CPD: chest pain duration; IC: informed consent; hs-TnT: high-sensitivity troponin T; mHS: modified HEART score.

From December 2011 to December 2013, a total of 1665 patients presenting to the emergency department were screened. Of them, 154 patients met the inclusion criteria and entered the analysis. The major reason for exclusion was late presentation, more than 6 hours from the beginning of chest pain, characteristic for the tertiary profile of enrolling center, and was observed in 995 patients (60%) (Figure 1).

Overall, AMI was diagnosed in 105 patients (68%) and UA in 30 patients (20%).

Patients were admitted with median delay of 4.1 hours from the onset of chest pain. Baseline profile was similar irrespective of the diagnosis of AMI (Table 2). Patients finally diagnosed with AMI had statistically higher baseline concentrations of hs-TnT (*p* < 0.001) and copeptin (*p* = 0.004) than other patients. There was no difference in GFR among the groups (*p* = 0.146). Statistically significant difference was found for admission ECG, which showed more frequent left bundle branch block in patients in whom AMI was diagnosed than in other patients (Table 2).

### 3.2. The Modified HEART Score

There were 44/154 patients (29%) classified as low-risk according to the mHS. The majority of patients were middle-aged, of moderate risk profile, low clinical suspicion of acute coronary syndrome, and with ST segment deviations on ECG. The distribution of the components of the mHS in the studied population is presented in Table 3.

### 3.3. Diagnostic Accuracy

In the studied population, the diagnosis of AMI was set in 68% of patients (105/154). The combination of hs-TnT, copeptin, and the mHS had higher sensitivity and NPV than hs-TnT alone (100%, 95% CI 96.6–100, and 100%, 95% CI 75.3–100, vs. 99.3%, 95% CI 88–97.9, and 85.4%, 95% CI 70.8–94.4, respectively) and higher than the combination of hs-TnT and the mHS (99.1%, 95% CI 94.8–100, and 94.4%, 95% CI 72.2–99.9) (Table 4). The accuracy of hs-TnT was 87%, hs-TnT with mHS 78%, and hs-TnT with copeptin and mHS 77% (Table 4).

### 3.4. Prognostic Accuracy

Overall, within 30 days from admission, the incidence of AMI and/or death of cardiovascular origin (including indexed AMI) was 69% (106/154 patients). Among patients ruled out by all three algorithms, event-free survival in patients stratified with single hs-TnT algorithm was 85% (35/41patients) and increased after additional use of the mHS (17/18 patients, 94%). The highest event-free survival was observed in patients stratified with hs-TnT, mHS, and copeptin algorithms (13/13 patients, 100%).

The lowest safety of rule out was observed for the algorithm of single hs-TnT (sensitivity 94.3%, 95% CI 88.1–97.9; NPV 85.4%, 95% CI 70.8–94.4) which was increased with additional use of mHS (sensitivity 99.1%, 95% CI 94.9–100; NPV 94.4%, 95% CI 72.7–99.9) and further with additional use of copeptin (sensitivity 100%, 95% CI 96.6–100; NPV 100%, 95% CI 75.3–100) (Table 4). Increasing prognostic safety of the algorithms was achieved at the expense of accuracy (88%, 79%, and 77%, respectively, Table 4).

Similar results were observed at one-year follow-up, completed in 147/154 (95%) patients. All three algorithms had lower accuracy than at 30 days. Safety of single hs-TnT was lower than that observed at 30 days and lower than that of hs-TnT/mHS and hs-TnT/mHS/copeptin (Table 4).

## 4. Discussion

According to current practice guidelines, it is highly recommended to assess prognosis in patients suspected for acute coronary syndrome [14]. Estimation of the probability of the development of adverse events in the short term after admission is crucial in regard to triage, diagnostic, and therapeutic decisions. Biomarker-based algorithms have a well-established role in this setting [1, 8, 14, 22]; however, high safety is achieved with serial sampling. Considering the constant need to shorten the time to diagnosis, several clinical scores, e.g., the modified HEART score, are under debate. This study is the first to evaluate safety of a strategy with single admission hs-TnT and copeptin sampling and the mHS in short-term prediction of adverse events in patients presenting with chest pain.

This study shows, as the major finding, that the algorithm combining admission concentration of hs-TnT and copeptin with the mHS is highly effective in estimation of short-term prognosis. High safety of ruling out adverse events within 30 days from admission (sensitivity 100%, NPV 100%) might enable early discharge of a subpopulation of patients who would undergo standard serial measurement. This approach might decrease the group who would undergo extended diagnostic process rather than be released home. The safety of the admission hs-TnT/mHS/copeptin algorithm was higher than that of single admission hs-TnT algorithm (5.7% difference in NVP and 14.6% difference in sensitivity) and the admission hs-TnT/mHS algorithm (0.9% difference in NVP and 5.6% difference in sensitivity). Of note, decisive information coming from the algorithm is available as soon as after the first blood sampling, before routine serial measurement. However, one should consider relatively small differences in NVP and sensitivity between the three algorithms. Thus, clinical relevance of the difference between hs-TnT/mHS and hs-TnT/mHS/copeptin should be interpreted with caution and might not justify the cost of a new laboratory test.

Standard cardiac troponin assays lacked sufficient safety when used alone. Copeptin and the HEART score improved markedly the evaluation of patients in combination with standard cardiac troponin by increasing prognostic value and leading to safe reduction in additional testing in emergency rooms [5, 23]. Introduction of high-sensitivity assays increased the diagnostic and prognostic value of cardiac troponin, decreasing the need for and simultaneously limiting the possibility of additional estimates for further improvement. This high level of safety was achieved at the expense of serial sampling which automatically prolonged the time spent to reach final decision. Single baseline hs-TnT concentration was postulated as a tool for diagnostic and prognostic purposes [6], but the accuracy of such approach was still suboptimal. To further shorten time to decision and maintain high safety presented by serial measurements, combined use of additional variables is being reassessed. According to recent studies, the use of baseline hs-Tn concentration below the limit of detection (LOD) and the mHS improved accuracy for short-term prognosis when compared to the score or troponin alone [24]. Even though lower than hs-TnT/mHS/copeptin, the hs-TnT/mHS algorithm still remains of better sensitivity/NPV than hs-TnT alone, thus reinforcing the interest for the mHS. Copeptin has an advantage over hs-TnT for long-term risk stratification [22] but has not been analysed neither with mHS nor hs-Tn for short-term risk. Combination of hs-TnT, copeptin, and the mHS, which represent different pathophysiological axes and carry complementary information on the origin and the character of chest pain and possible myocardial ischemia, results in an algorithm of excellent prognostic performance. As an effect, we get a tool for rapid and firm rule out with high safety, comparable to that achieved after serial hs-Tn sampling, but in shorter time. Thus, the need for further serial measurements is eliminated in the subgroup of ruled-out patients, who would be possibly ruled out also with one of the recommended currently serial hs-Tn protocols. Observed drop in accuracy for the studied algorithm compared to single hs-TnT algorithm (9% after adding the mHS to hs-TnT and further 2% after adding copeptin) would have little clinical relevance if the proposed rule-out algorithm was considered as a screening for inclusion for serial sampling rather than a substitute, so that patients not rule out at baseline with proposed algorithm would be qualified for current standard of care and undergo serial sampling.

A great advantage of the algorithm is, first, the availability of all data at baseline and, second, the ease of assessment and interpretation by involving well-known variables with firm clinical experience in the interpretation of all of them alone.

Further studies comparing the hs-TnT/mHS/copeptin algorithm with currently recommended short diagnostic protocols with serial measurements of hs-TnT are needed before wider clinical application of the proposed algorithm.

In conclusion, the combination of baseline hs-TnT, copeptin, and the mHS has an excellent sensitivity and NPV for short-term risk stratification. Considering an inescapable concomitant decrease in efficacy, it should be implemented carefully, but such approach might improve the triage system in emergency departments and be a bridge for inclusion to serial blood sampling algorithms.

## 5. Limitations

The presented study was a secondary analysis of the COPACS study, designed to assess the diagnostic and prognostic role of copeptin in patients presenting to the ED with chest pain and share the limitation of the COPACS study [9, 22]. A priori established inclusion and exclusion criteria as such might bias the results. First, the small study sample of patients presenting with chest pain with high percentage of diagnosed AMI might affect internal as well as external validity of the study and clinical application of the results. Thus, conclusions should be interpreted accordingly. Second, the time from the onset of chest pain to blood draw for analysed biomarkers in the current analysis might differ from that observed in clinical practice, resulting in possible differences in sensitivity of tested algorithms. Further, the results cannot be interpolated to other troponin or copeptin assays and similar analysis with the use of different assays should be conducted to obtain more evidence on possible practical utility of the proposed algorithm.

## Figures and Tables

**Table 1:** Composition of the modified HEART score.

Age	Electrocardiogram	Risk factors^∗^	Clinical presentation^†^	Points
≥65 years	ST segment depression/elevation	≥3 risk factors or history of CAD^∗∗^	High suspicion (3/3 criteria)	2
45–64 years	LBBB and LVH	1 or 2 risk factors	Moderate suspicion (2/3 criteria)	1
<45 years	Nonspecific changes	No risk factors	Low suspicion (0–1/3 criteria)	0

CAD: coronary artery disease; LBBB: left bundle branch block; LVH: left ventricular hypertrophy. ^∗^Considered risk factors: hypertension, diabetes mellitus, and smoking history. ^∗∗^History of coronary artery disease included the history of myocardial infarction, cardiac revascularization, or former diagnosis of coronary artery disease. ^†^Considered criteria: type of chest pain claimed as pressure, aggravation of chest pain with physical activity, and radiation to arms and/or shoulders.

**Table 2:** Baseline characteristics.

	All patients *n* = 154	AMI *n* = 105	No AMI *n* = 49
Age (years)	63 (57–73)	63 (57–73)	61 (55–71)
Female sex	54, 35%	32, 30%	22, 45%
Time since the onset of chest pain (hours)	4.1 (3.0–5.9)	4.2 (2.8–5.7)	4.1 (3.0–6.0)
hs-TnT (ng/l)	33 (13–143)	80 (31–210)	9.1 (6.7–14)
Copeptin (pmol/l)	12 (5.7–21)	14 (6.4–27)	9.0 (4.0–14)
GFR (ml/min)	92 (76–110)	90 (70–108)	95 (80–113)
*ECG pattern*			
ST deviation	95, 62%	70, 67%	25, 51%
LBBB	10, 6.5%	10, 9.5%	0, 0%
LVH	14, 9.1%	11, 10%	3, 6%
*Risk factors*			
Hypertension	114, 74%	84, 80%	30, 61%
Diabetes	42, 27%	33, 31%	9, 18%
Smoking	82, 53%	60, 57%	22, 45%
History of CAD^∗^	67, 44%	43, 41%	24, 49%

Data are presented as median (25th–75th percentile) or *n*, %. AMI: acute myocardial infarction; LBBB: left bundle branch block; LVH: left ventricular hypertrophy; CAD: coronary artery disease. ^∗^History of CAD included the history of myocardial infarction, cardiac revascularization, or former diagnosis of coronary artery disease.

**Table 3:** Modified HEART score: distribution of the components.

Characteristic	Outcome
Age	
≥65 years	68; 44%
45–64 years	81; 53%
<45 years	5; 3%
Electrocardiogram	
ST depression/elevation	95; 62%
LBBB and LVH	14; 9%
Nonspecific changes	45; 29%
Risk factors^∗^	
≥3 risk factors or history of CAD^∗∗^	18; 12%
1 or 2 risk factors	121; 79%
No risk factors	15; 10%
Clinical presentation^†^	
High suspicion (3/3 criteria)	4; 2%
Moderate suspicion (2/3 criteria)	41; 27%
Low suspicion (0–1/3 criteria)	109; 71%

Data are presented as *n*; %. LBBB: left bundle branch block; LVH: left ventricular hypertrophy; CAD: coronary artery disease. ^∗^Considered risk factors: hypertension, diabetes mellitus, and smoking history. ^∗∗^History of CAD included the history of myocardial infarction, cardiac revascularization, or former diagnosis of coronary artery disease. ^†^Considered criteria: type of chest pain claimed as pressure, aggravation of chest pain with physical activity, and radiation to arms and/or shoulders.

**(a):** 

	Diagnosis of AMI		
	hs-TnT^∗^	hs-TnT^∗^with mHS^∗∗^	hs-TnT^∗^ with copeptin^†^ and mHS^∗∗^
Sensitivity	99.3%	99.1%	100%
95% CI (%)	88–97.9	94.8–100	96.6–100
NPV	85.4%	94.4%	100%
95% CI (%)	70.8–94.4	72.2–99.9	75.3–100
Test accuracy	134/154 (87%)	121/154 (78%)	118/154 (77%)
*p* value	<0.001	<0.001	<0.001

**(b):** 

	AMI/death at 30 days		
	hs-TnT^∗^	hs-TnT^∗^ with mHS^∗∗^	hs-TnT^∗^ with copeptin^†^ and mHS^∗∗^
Sensitivity	94.3%	99.1%	100%
95% CI (%)	88.1–97.9	94.9–100	96.6–100
NPV	85.4%	94.4%	100%
95% CI (%)	70.8–94.4	72.7–99.9	75.3–100
Test accuracy	135/154 (88%)	122/154 (79%)	119/154 (77%)
*p* value	<0.001	<0.001	<0.001

**(c):** 

	AMI/death at one year		
	hs-TnT^∗^	hs-TnT^∗^ with mHS^∗∗^	hs-TnT^∗^ with copeptin^†^ and mHS^∗∗^
Sensitivity	92.2%	99%	100%
95% CI (%)	85.1–96.6	94.7–100	96.5–100
NPV	80.5%	94.4%	100%
95% CI (%)	65.1–91.2	72.7–99.9	75.3–100
Test accuracy	127/147 (82%)	118/147 (80%)	115/147 (75%)
*p* value	<0.001	<0.001	<0.001

Data are presented as median (25th–75th percentile) or *n* (%). hs-TnT: high-sensitivity troponin T; mHS: modified HEART score; AMI: acute myocardial infarction; CI: confidence interval; NPV: negative predictive value. ^∗^Baseline hs-TnT rule out at 14 ng/l. ^∗∗^mHS rule out at ≤3. ^†^Baseline copeptin rule out at 17.4 pmol/l.

## Data Availability

All data are stored in a form of an electronic database together with results from analysis in a form of a statistical software report.
